# Does spirituality and religiousness influence verbal functions in stroke patients?

**DOI:** 10.1192/j.eurpsy.2021.1140

**Published:** 2021-08-13

**Authors:** V. Giannouli, K. Giannoulis

**Affiliations:** 1 Institute Of Neurobiology, Bulgarian Academy of Sciences, Sofia, Bulgaria; 2 Faculty Of Theology, Aristotle University of Thessaloniki, Thessaloniki, Greece

**Keywords:** Spirituality, verbal functions, stroke, religiousness

## Abstract

**Introduction:**

Patients after stroke may experience different cognitive and emotional changes based on the levels of their spirituality and religiousness.

**Objectives:**

This preliminary study aims to explore whether self-reports in two questionnaires measuring the personal experience of spirituality and religiousness can influence cognition and more specifically performance on neuropsychological tests examining verbal functions.

**Methods:**

Fifteen male stroke patients participated voluntarily one year after their hospitalization. The mean age of the patients was 75.58 years (SD = 7.50, range 61-90), level of education 15.47 years (SD = 3.82). In addition to that, fifteen controls with similar demographics, free of physical and mental diseases, were also examined. Depressive symptoms of the participants were assessed with the 15-item Geriatric Depression Scale. The Daily Spiritual Experience Scale, the Systems of Belief Inventory (SBI-15R) and a number of standardized tests examining verbal functions such as word list learning (number of words on immediate and delayed recall), story learning (number of words on immediate and delayed recall).

**Results:**

showed a statistically significant difference between the control group and the stroke group in performance regarding verbal functions, with the first group showing higher scores. No statistically significant difference was found between the two groups regarding the levels of spirituality and religiousness.

**Conclusions:**

Although spirituality and religiousness may be related with quality of life, cognitive functions such as verbal functions are not influenced one year post-stroke.

